# Physical Activity, a Critical Exposure Factor of Environmental Pollution in Children and Adolescents Health Risk Assessment

**DOI:** 10.3390/ijerph15020176

**Published:** 2018-01-22

**Authors:** Jingmei Dong, Su Zhang, Li Xia, Yi Yu, Shuangshuang Hu, Jingyu Sun, Ping Zhou, Peijie Chen

**Affiliations:** 1 Department of Physical Education, Tongji University, Shanghai 200092, China; djm1969@tongji.edu.cn (J.D.); zhangsu13@126.com (S.Z.); 18801765816@163.com (L.X.); yuyi25123@126.com (Y.Y.); 1731775@tongji.edu.cn (S.H.); jingyusun@126.com (J.S.); Hizhouping23@163.com (P.Z.); 2 Department of Sports Science, Shanghai University of Sport, Shanghai 200438, China

**Keywords:** air pollution, children and adolescents, physical activity, environmental health risk assessment, exposure factor

## Abstract

It is an extremely urgent problem that physical fitness promotion must face not only the increasing air pollution but also the decline of physical activity level of children and adolescents worldwide at present, which is the major reason that forms an inactive lifestyle and does harm to adolescents’ health. Thus, it is necessary to focus on the exposure factor in environmental health risk assessment (EHRA) which conducts supervision of environmental pollution and survey of adolescents’ activity patterns according to the harmful characteristics of air pollutant and relationship between dose and response. Some countries, such as USA, Canada and Australia, regard both respiratory rate and physical activity pattern as main exposure factors for adolescents in both air pollution health risk assessment and exercise risk assessment to forecast a safe exposing condition of pollutant for adolescents while they are doing exercise outdoors. In addition, it suggests that the testing indexes and testing methods of these two exposure factors, such as investigating the time of daily physical activity, strength, and characteristic of frequency, help to set up the quantitative relationship between environmental pollution index and the time, strength, frequency of daily activities, and formulate children’s and adolescents’ activity instructions under different levels of environmental pollutions. As smog becomes increasingly serious at present, it is meaningful to take physical activity as a critical composition of exposure factor and establish physical activity guideline, so as to reduce the risk of air pollution, and promote physical health of children and adolescents effectively.

## 1. Introduction

Adolescence is a significant transitional stage from childhood to adulthood. Physical activity in adolescence contributes to healthy adulthood lifestyle, and reduction of chronic disease incidence [1]. Human body has evolved in such a way that most of its systems (e.g., skeletal, muscle, metabolic, and cardiovascular) do not develop and function in an optimum way unless they are stimulated by frequent physical activities [2]. The physical fitness level in adolescence is associated with health-related outcomes, such as risk of obesity and cardiovascular diseases, skeletal health and mental health [3,4]. There are many debates on whether physical fitness level has declined in the last 30 years. Evidence suggests that there has been a decline of physical activity among children and adolescents in many countries [5,6]. The decrease of physical activity, especially outdoor physical activity leads to the decline of children’s physical fitness level. Many domestic and international studies show that lack of physical activity may be an important factor leading to the decline of physical fitness level [7]. Daily physical activity level and participation of sporting activity are closely related to physical fitness level. An analysis of the report on physical health factors of adolescents in Shanghai shows that lack of physical activity is an important factor of the decline of students’ physical fitness level [8].

Public health organizations and schools make efforts to increase physical activity level and decrease sedentary time, but, at the same time, environment contaminant should also be considered because of the increasingly serious environmental pollution in recent years in the world, especially in China [9]. Since air pollution exposure is associated with physical activity and performance status to a great extent, it has enormous potential to harm health when children grow up [10,11], but there are few relevant studies to explore the role of environmental factor in physical activity and the harm of exposure to air pollution while children are doing exercise. Because of air pollution, physical education and outdoor activities of primary and secondary schools are sometimes cancelled in Beijing and Hangzhou, which causes a more severe situation of physical health of adolescents. Consequently, physical activity of adolescents shows an ever decreasing trend because students’ outdoor activities have to be reduced.

Considering these two facts, in this article, the concept and study methods of inhalation exposure factor are introduced; the status of inhalation exposure factor of youth in China and the relationship between exposure factor and physical activity of youth in haze pollution in urban area are analyzed. Professional guidance can offer advice to physical activity and emergency plan, which helps to alleviate and avoid health hazards of haze pollution during outdoor activities. It is necessary to introduce exposure factor from environmental health risk assessment (EHRA), and to understand the composition of exposure factor related to physical activity in environmental health, so as to establish physical activity guideline to reduce the risk of physical activity in polluted environment.

## 2. Physical Activity, Environmental Pollution and Heath of Adolescents

### 2.1. Global Adolescents’ Physical Activity Level and Fitness

Since the industrial revolution, the development of new technologies has enabled people to reduce the amount of physical activity to accomplish many tasks in their daily lives. In 2017, the World Health Organization (WHO) published 19 leading lethal factors, among which, physical inactivity and urban outdoor air pollution are ranked 3rd and 11th, respectively [12]. In addition, a survey made by WHO in 2017 showed that 81% of school-going adolescents aged 11–17 in the world did not do sufficient physical activities. They do not do 60-min moderate-to-vigorous physical activity every day, as recommended by WHO [13].

According to the report of Lancet (Pedro et al., 2012) [14], the data of adolescents’ (13–15 years old) physical activity level of 105 countries indicate that 80.3% (80.1–80.5%) of adolescents are physically inactive, which is higher in girls than in boys, and the percentage is still rising in high-income countries [6]. The proportion of 9–17-year-old adolescents who do less than 60 min of physical activity of moderate-to-vigorous intensity per day is 91.6% in boys and over 98.1% in girls in China [15]. Publicly available data from the Health Behavior in School-Aged Children (HBSC) [16] reports and available raw data [17,18] of 38 European countries indicate that reductions in physical activity have been recorded. The recordings involve children and adolescents aged from 8 to 18, from 1998 to 2010, in American and European countries [19,20]. Conversely, a study in Sweden [21] shows that sedentary behavior has become a kind of normal lifestyle. Boys are more active than girls. Thus, from these data, we can see a decline of physical activity level among children and adolescents globally. That is to say, higher level of physical activity is associated with better physical, social and psychological health status of young people. Physical activity is inversely correlated with overweight and obesity disease. The study of students’ fitness indicates that the fitness status of adolescents is in a worrisome situation recently.

### 2.2. Air Pollution and Children’s Health

Environment plays an important role in the health of population. The database established by WHO in 2014 contains the results of ambient air pollution monitoring from almost 1600 cities in 91 countries. It finds that half of the cities’ air pollution level is at least 2.5 times higher than the WHO standard [22]. Physical inactivity and urban outdoor air pollution have become global problems. According to WHO’s data, approximately 25% of preventable illness in the world is attributable to environment; it also reflects that China’s annual burden of disease attributed to environmental factors is 21%, which is 8% higher than the Unite States’ [23,24]. According to the Global Burden of Disease Study in 2010, ambient particulate matter pollution (PM_2.5_) is the 4th leading lethal factor in China [25]. In 2012, the Environmentally Sustainable Future: Country Environmental Analysis of the People’s Republic of China released the world’s 10 most serious air pollution cities, of which seven are in China [26]. Thus, WHO proposed a new concept of large health named “six-dimensional view of health”; the new concept of health includes physical health (science diet and balanced nutrition), psychological health, intellective health, mental health, social health and environmental health.

Environmental health is proposed in the presupposition of the worsening global environment. Environmental health has played an important role in human health, particularly in the face of terribly polluted environment in our cities [27]. With the growing proportion of environmental factors affecting health of population, and the increasing occurrence rate of various types of environmental health damaging incidents, the problems of environment and health have gradually attracted the concern of health researchers and government. More profound correlative studies are conducted; the assessment of health risk is closely related to the environment.

However, physical inactivity and exposure to air pollution are important risk factors of death and disease globally. Taking these limitations into account, the reported findings are consistent with several other recent studies which also use objective methods to measure physical activity. In addition, the level of total physical activity and moderate-to-vigorous physical activity observed in a recent study are similar to those observed in the United Kingdom that both younger and older samples exhibit sedentary behavior [28].

## 3. Exposure Factor in Environmental Health Assessment

Risk assessment is the process of estimating the potential impact of a chemical, physical, microbiological or psychosocial hazard on a specific human population or ecological system under a specific set of conditions and for a certain time frame. The scope of environmental health risk assessment (EHRA) can include the following aspects: chemical pollutants and contaminants in air, water and soil; pathogenic microbiological contaminants in food and water; radiation source electromagnetic fields (EMFs); and climate and climate change [29]. Thus, EHRA is an evaluation method which connects environmental pollution with human health, by estimating the probability of adverse effects to humans to evaluate the impact on human health exposing to this factor. The application of this factor is to take a degree of risk as the evaluation index, link the level of environmental pollution to human health, and quantitatively describe the hazards of pollutants on human body [30].

EHRA is based on the relatively clear understanding of pollutants’ hazardous properties and the dose-response relationship, through monitoring the results of environmental exposure concentrations and the investigations of the behavior of population exposure, following a specific procedure of an assessment model to predict health risks of the pollution in the specific situation. Exposure parameter (exposure factor) is the key parameter to determine the exposure factor and health risks of environmental pollution [31]. It is used to describe the parameters of behavior and character of human body through the respiratory tract, gastrointestinal tract, and skin exposure to environmental pollutants. Therefore, the exposure parameters include respiratory exposure parameters, oral exposure parameters, skin exposure parameters and behavioral activity patterns of exposure parameters. Respiration rate and activity patterns are major inhalation exposure parameters in health risk assessment of air pollution [32].

Exposure factors are factors related to human behavior and characteristics that help determine an individual’s exposure to an agent. Those aspects of human health are determined by physical, chemical, biological and social factors in the environment. Environmental health practice includes assessment, correction, control and prevention of environmental factors, which can adversely affect health; accordingly, the enhancement of those aspects of environment can improve human health. Human bodies are exposed to environmental pollutant mainly through respiratory tract, ingestion, or skin [32]. Thus, according to different pathways, exposure parameters can be divided into inhalation exposure factors, ingestion exposure factors, dermal exposure factors and basic parameters applicable to all pathways. Each kind of exposure factor includes intake rate, activity patterns and other parameters. Since children may be at higher risk of adverse health than adults when they are exposed to toxic pollutants in air, food, and soil—due to their smaller, developing bodies, and their natural instinct of prolonged contact with soil and dust—a child-specific exposure factors handbook has been created by the U.S. Environmental Protection Agency [33].

## 4. Inhalation Exposure Factors of Children

Ambient and indoor air is a potential source of children’s exposure to toxic substances. Children are exposed to contaminated air during a variety of activities in different environments. Children may be exposed because of the sources which bring pollutant to ambient air. Children may also inhale chemicals from the indoor use of various consumer products. Due to their size, physiology, and activity level, the inhalation rates of children are different from adults. Inhalation exposure factor is average daily dose of the exposure to pollutants through respiratory tract of human. Formula (1) is (Li et al., 2012) [34]:

(1) $$ADD=\frac{C\times IR\times ET\times EF\times ED}{BW\times AT}$$

In Formula (1), *ADD* is average daily dose of the exposure to a compound by breathing (mg/(kg·d)). *C* is mass concentration of the compound in ambient air (mg/m^3^). *IR* is inhalation (respiration) rate (m^3^/d). *ET* is exposure time per day (h/d). *EF* is exposure frequency (d/a). *ED* is exposure duration (a); it is the specified units of time exposing to contaminants. *BW* is body weight (kg). *AT* is average time of exposure (h). Some explanation of the units is needed. “h” means hour; “d” means day; and “a” represents a time unit or means a certain period, which is specified by the researchers. Taking carcinogens for example, the duration of a lifetime has traditionally been assigned the nominal value of 70 years as a reasonable approximation. The lifetime of exposures is 70 a × 365 d/a. For acute exposures, the doses are usually averaged over a day or a single event.

### 4.1. Methods of Measuring Inhalation Rate

There are three methods of measuring inhalation rate: direct measurement, heart-rate-inhalation-rate regression and body energy metabolism. Direct measurement means that inhalation rates at various activity levels are directly measured by devices such as spirometer and collection system. However, it is relatively cumbersome, thus not suitable for large-scale studies [27].

Heart-rate-inhalation-rate regression is to select a representative sample in the process of measuring inhalation rates and heart rates, establish simple or multiple liner regression models, and then to calculate inhalation rates according to heart rates of all kinds of people. This method will be better for large-scale surveys if samples are selected properly. Body energy metabolism means that inhalation rates are determined according to the consumption of energy and oxygen per unit time, which refers to one day or the time spent on one type of activity. Advantages of the method include that the calculation is simple, and it is easy to obtain data. The disadvantage is that the accuracy needs to be improved [27]. It is widely used by researchers at home and abroad [34].

### 4.2. Data Collection of Physical Activity Patterns

Physical activity (PA) is defined as any body movement caused by skeletal muscles and resulting in energy expenditure. Therefore, PA includes not only the traditional sense of physical exercise (such as running, swimming, and ball games), but also daily life physical activity (such as walking up and down stairs, and doing housework). It is generally described by intensity, frequency, duration and type of activity. PA can be divided into two categories: baseline activity and health-enhancing physical activity. Baseline activity refers to the light-intensity activity of daily life, such as standing, walking slowly, and lifting lightweight objects. Health-enhancing physical activity is the activity whose physical activity level is above the baseline; it will provide health benefits. Brisk walking, jumping rope, dancing, lifting weights, climbing on playground equipment at recess, and doing yoga are all examples of health-enhancing physical activities [35].

According to the report of WHO since 2008, physical activity data are generally obtained through the recall of questionnaires and journals recording human activities and microenvironment [17]. To assess exposure factor and establish data models, U.S., Canada and other countries have developed large-scale activity pattern surveys mainly including the California Study of Children’s Activity Patterns Survey (CAPS), National Human Activity Pattern Survey (NHAPS) and the Canadian Human Activity Pattern Survey (CHAPS). The contents of these three surveys are listed in Table 1. As shown in Table 1, the use of 24-h retrospective diaries is the major method of recording physical activity patterns. The test time of physical activity does not cover 24 h and the sample is not large enough, except NHAPS at early stage (1992–1994). However, the scale becomes larger and global positioning system (GPS) can provide personal information in recent years [36].

## 5. Relationship between Physical Activity and Inhalation Exposure Factor of Children and Adolescents

### 5.1. Relationship between Physical Activity and Inhalation Rate

Inhalation rate for determining intake of air pollutant must be estimated generally, because it is difficult to make direct inhalation measurement in free-standing population [37]. According to body energy metabolism, the calculation of inhalation rate is shown in Formula (2) [37]:

(2) IR=E×H×VQ

*H* is the volume of oxygen consumed to produce 1 kcal of energy (m^3^/kcal, L/kcal or L/KJ), which takes 0.05 L/KJ here. *VQ* ratio is the volume of air to the volume of oxygen breathed per unit time (non-dimensional), which generally takes 27. The calculation of *E* is shown in Formula (3):

(3) E=BMR×N

*BMR* is the minimum amount of required energy to support basic cellular respiration during rest or non-active digestion (kJ/d or MJ/d); and *N* ratio is the consumption of energy on the activity level of *BMR* (non-dimensional).

In general, *BMR* is calculated by models [38]. Shizgal–Rosa formula is suitable for estimating *BMR* of people under the age of 18 [39]. Formulae (4) and (5) are, respectively, used to calculate *BMR* of males and females under the age of 18:

(4) BMR=370+20H+52BW−25A

(5) BMR=1873+13H+39BW−18A

*H* is the height (cm); *BW* is the body weight (kg); and *A* is the age (a).

According to Formulae (1)–(5), the following expression can be derived:

(6) $$ADD=\{\begin{array}{c} \frac{1.35\times C\times \left(370+20H+52BW-25A\right)\times N\times ET\times EF\times ED}{BW\times AT}\\ \frac{1.35\times C\times \left(1873+13H+39BW-18A\right)\times N\times ET\times EF\times ED}{BW\times AT} \end{array}$$

According to Formula (6), *C*, *H*, *BW*, *A* and *AT* are uncontrollable factors, while *N*, *ET* and *ED* are controllable factors: *N* is related to the intensity of physical activity; *ET* and *ED* are exposure time/duration, where the period used is the actual period of exposure in outdoor situation, both associated with the time of physical activity; and *EF* is associated with the frequency of physical activity. Under certain circumstances, *C*, *H*, *BW*, *A* and *AT* are settled; the larger *N*, *ET* and *ED*, the larger the value of the *ADD* and vice versa. Consequently, in certain circumstances, with a given amount of pollution, height, weight and age of children and adolescents, the greater the intensity of physical activity, the longer the time, the higher the frequency, and the larger the value of daily exposure dose.

### 5.2. Research on Inhalation Rate in Different Physical Activities

The consumption of energy in resting state equals the basal metabolic rate (*BMR*), while sedentary, light intensity physical activity, moderate intensity physical activity, high intensity physical activity and extremely high intensity physical activity are about 1.2, 1.5, 4, 6 and 10 times of *BMR*, respectively [40].

Therefore, we made the multi-factor analysis of variance of the inhalation rate in different ages, genders, and levels of physical activities. We collected the data of physical supervision of 6–20-year-old children and adolescents in Shanghai in 2014 [41], and calculated the basal metabolic rate to verify the inhalation rate of children and adolescents in different levels of physical activities in these ages. The result shows that the inhalation rate of male and female children and adolescents in Shanghai in different physical activities are about 0.221–2.432 m^3^/h and 0.238–1.969 m^3^/h, respectively.

**Figure 1:**
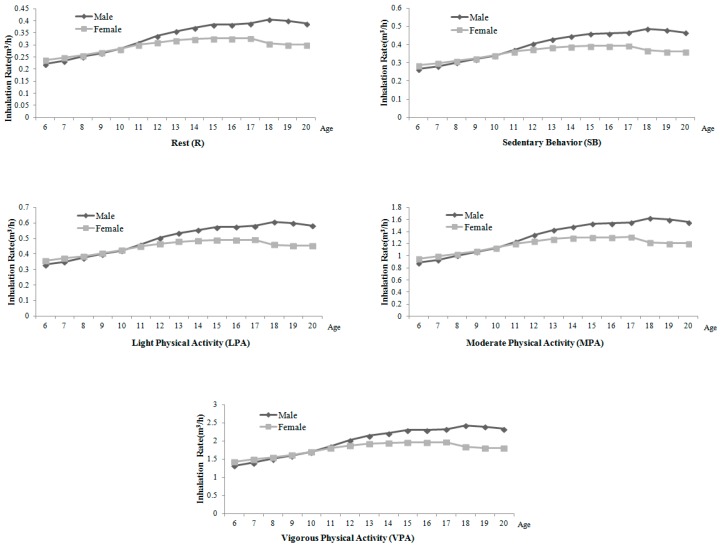
Change characteristics of inhalation rate of children and adolescents of different ages and genders in Shanghai in different physical activities.

As illustrated in Table 2 and Figure 1, the inhalation rate of 6–20-year-old children and adolescents in Shanghai has the following characteristics: (1) Generally speaking, the inhalation rate in rest, sedentary behavior, light physical activity, moderate physical activity and vigorous physical activity increase with age. (2) However, the Puberty Cross phenomenon appears at the age of 10, which has the following specific performances: before the age of 10, the inhalation rate of female children and adolescents is higher than males of the same age, but, after 10, the inhalation rate of males is higher than females’ of the same age, and the gap becomes wider with age. It is related to the asynchronized development step of males and females in puberty [42]. Males and females enter puberty at different ages. Girls’ puberty is earlier than boys’: girls’ growth spurt is usually before the age of 10, while boys’ is always after 10 years old. Boys stop growing later and have a longer growing period, so the growing and metabolic level of boys will be significantly higher than girls of the same age, and the gap would be gradually expanded. That is to say, in Shanghai, under the same level of physical activity, there are different inhalation rates of children and adolescents because of gender and age variation; the inhalation rate of children and adolescents is influenced by age, gender, activity intensity and other factors.

**Figure 2:**
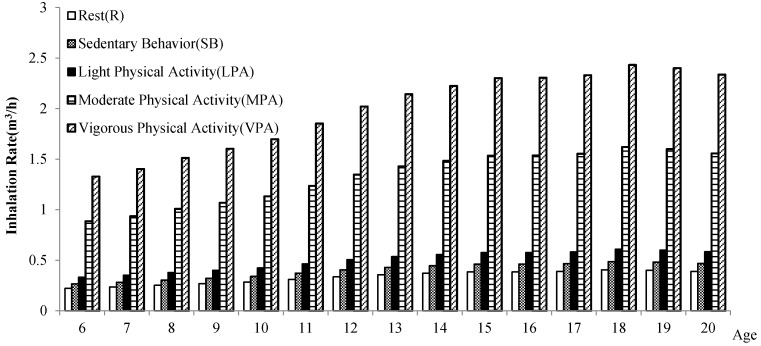
Comparison of the inhalation rate of children and adolescents in Shanghai in different physical activities (male).

**Figure 3:**
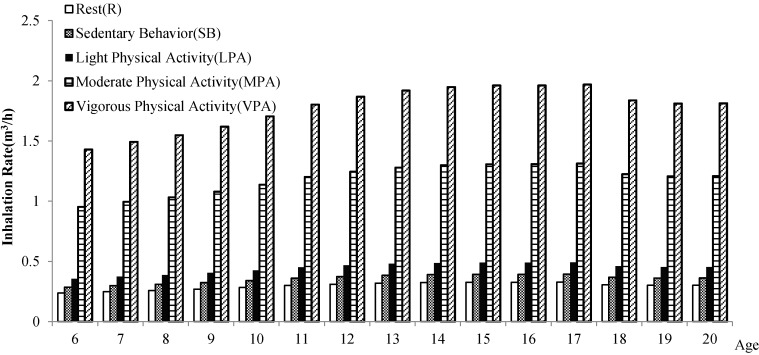
Comparison of the inhalation rate of children and adolescents in Shanghai in different physical activities (female).

At the same age and gender (see Figure 2 and Figure 3), children and adolescents in different levels of physical activity have different inhalation rates, which shows a decreasing trend: the inhalation rate (*IR*) of vigorous physical activity > *IR* of moderate physical activity > *IR* of light physical activity > *IR* of sedentary behavior > *IR* of rest. It indicates that the higher the level of physical activity, the higher the short-term inhalation rate.

The inhalation rate is different in different levels of physical activity. Yan Yang’s (2004) and Zongshuang Wang’s (2009) [43,44] researches on adults also show the gender variance and age variance of inhalation rate in different physical activities. Therefore, in the process of environmental health risk assessment of air pollution, neglecting the difference in gender, age and physical activity level will cause unreasonable evaluation, increase the uncertainty of assessment result [45], or even lead to errors of assessment for special populations and the occurrence of accidents [46,47].

## 6. Status of Children and Adolescents Respiratory Exposure Parameters in the World

### 6.1. Exposure Parameters in the World

The United States is the first country publishing a database and manual of exposure parameters in the world. The first edition of the “Manual Exposure” was published in 1989 [48], and later revised in 1997 and 2011. In addition, a series of companion manuals of Exposure Factors Handbook have been issued, such as Social Demographic Data [49], Options for Development of Parametric Probability Distributions for Exposure Factors [50] and Food Intake Distributions [51]. These handbooks have been widely cited to research health risk assessment and management.

The Japanese Exposure Factors Handbook was compiled in 2007 by National Institute of Advanced Industrial Science and Technology (AIST). It includes parameters of human body characteristics (body weight, life expectancy, surface area, etc.), exposure factors through the mouth, dermal exposure factors, time-activity patterns, etc. [52]. However, unlike the U.S., this book also includes exposure concentration of chloroform, benzene, toluene, etc.; the body burden of dioxins, cadmium and mercury in mother’s milk, blood, urine and hair; etc.

Based on the characteristics of the Korean residents, the Korean Exposure Factors Handbook was compiled in reference to the framework of the U.S. [53].

The Australian Exposure Guidance Handbook was published in 2012 and was prepared as a companion of the health guidance document updated in 2012, Environmental Health Risk Assessment: Guidelines for Assessing Human Health Risks from Environmental Hazards [54].

The Exposure Factors Handbook of Chinese Population was issued in 2013 by the Ministry of Environmental Protection of China [55]. It is the first exposure factors handbook of China, which is applied to the derivation of environmental baseline, prevention and control of prioritizing pollution, environmental impact assessment, risk management of chemicals and risk assessment of contaminated sites. Besides, the companion manual the Report of Environmental Exposure Related Activity Patterns Research of Chinese Population was issued [56]. It reflects the characteristics of Chinese population, greatly improved the accuracy of environmental health risk assessment, and promoted the development of evaluation of defense line of environmental health in China.

Currently, the exposure parameters manual of the United States, Japan and Canada all include weight, life expectancy, respiration rate, water rate, skin surface area, the exposure of food, the average exposure time and other major exposure parameters. They take into account the exposure in different media, in different countries, and combine their own ethnic characteristics.

### 6.2. Inhalation Exposure Factors of Chinese Children and Adolescents

A review shows that 42 papers published in English have summarized potential factors which can influence physical activity of Chinese children and adolescents [57]. However, there is no large-scale survey that can provide some references for children inhalation exposure factors. There are no systematic reports of activity patterns of children and adolescents in China, except a few reports on exposure time. A children’s exposure factors handbook has not yet been released in China. We mainly refer to exposure factors of children in the United States, Japan and other countries in health risk assessment of air pollution. Due to different activity patterns influenced by races, habits and customs, inhalation exposure factors of children abroad cannot be based on the characteristics and behaviors of the exposure factors in China. Thus, the reference of oversea inhalation exposure factors tends to make great error.

The 2012 China Health Statistics Yearbook contains lifetime expectancy of males and females, regional lifetime expectancy and regional structure of population age [57]. In 2010, the third national health research was jointly organized by 10 departments (Administration of Sports, Ministry of Education, Ministry of Science and Technology, etc.) in 31 provinces, autonomous regions and municipalities, which include Chinese citizens aged 3–18 years old [58]. Body shape was one of the test indexes. Much information of the research can be used as the basis of inhalation rates data of children and adolescents, but we are still far away from getting children inhalation rates.

### 6.3. Features of Inhalation Exposure Factors of Chinese Children and Adolescents

The inhalation rates used to support such studies depend on the attributes of exposed population (e.g., age, gender, occupational status, etc.) and, more importantly, the average time used to characterize the contaminant concentration in air and the division of long-term period (the daily inhalation rates) and short-term period (time inhalation rates). Long-term inhalation rates for adults and children (including infants) are presented as daily rates (m^3^/d). Short-term exposure is repeated exposure for more than 24 h, up to 30 days. Short-term inhalation rates are reported for adults and children (including infants) doing various activities in m^3^/min. Thus, the inhalation rates are gained by Formulae (2)–(5). The methodology presented here for simulating daily average energy expenditures and related inhalation rates is a hybrid approach that utilizes data of body weight derived from national health and nutrition examination surveys (NHANES, 2014) [59].

Based on the methods above, inhalation rates and activity patterns of Chinese children and adolescents are calculated and briefly summarized in Table 3. As shown in Table 3, with the growth of children and adolescents, long-term inhalation rates of both boys and girls show a gradually increasing trend. At the age of 15–18, the inhalation rates of both genders reach the maximum, 13.5 m^3^/d and 10.8 m^3^/d respectively. Meanwhile, the inhalation rates of boys are not entirely higher than girls. Before six years old, girls’ inhalation rates are higher than boys’ by 8.5–14.9%. After six years old, boys’ inhalation rates are higher than girls’ by 11.6–25.0%.

A difficulty of estimating average inhalation rates of dichotomous active/non-active periods is the omission of elevated inhalation rates that occur as people engage in a variety of physical activities. As shown in Table 4 the activity is divided into six types according to energy expenditure: rest, sedentary, light intensity, moderate intensity, high intensity and extremely high intensity [60]. For the convenience of comparison, the group is divided into the group of children (younger than six years old) and the group of adolescents (older than six years old and younger than 18 years old).

**Figure 4:**
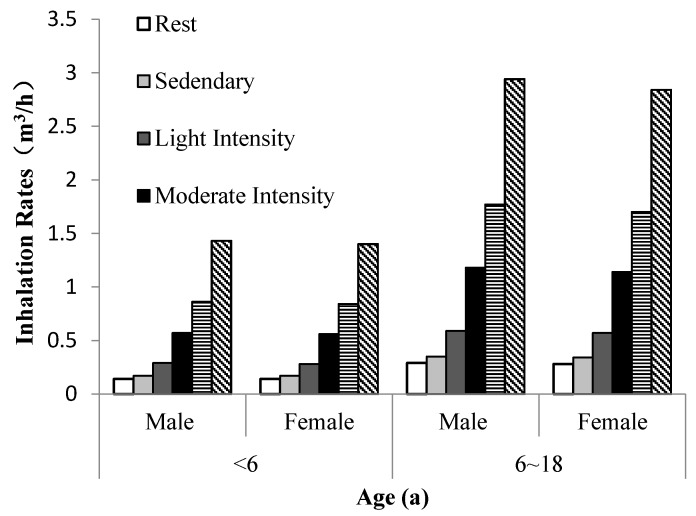
Short-term inhalation rates at different activity levels of Chinese children and adolescents.

When activity levels are different, average inhalation rates change. Thus, intake dose of pollutants will be affected. As shown in Table 3, Figure 1 and Figure 4, inhalation rates go up with the increase of activity level. The inhalation rates of both boys and girls in extremely high activity level are higher than those in high, moderate and light activity levels. Therefore, when we conduct air pollutants exposure and health risk assessment, a certain degree of error will be made if the differences of activity levels are ignored.

### 6.4. Comparison of Inhalation Exposure Factors of Children and Adolescents in the World

**Figure 5:**
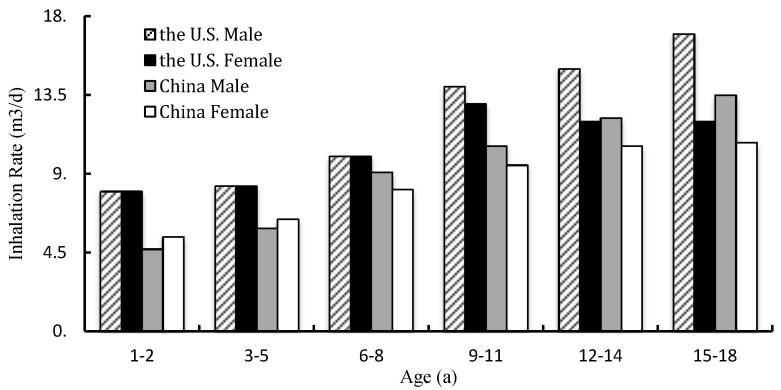
Comparison of long-term inhalation rates of children between the U.S. and China.

Time-activity patterns of different age groups in the U.S. and China are illustrated in Figure 5. Almost all kinds of time–activity patterns of all ages (from one year old to eighteen years old) have been studied. Inhalation rates of Chinese children of all age groups are lower than the United States. For boys, the rates are lower than the United States by 9.0–41.3%. For girls, the counterpart value is lower by 10.0–32.5%.

Considering the comparative analysis above, we can see that inhalation exposure factors of children and adolescents of these countries are different because of the difference of race, geography, socioeconomics, etc. Thus, when we assess inhalation exposure rates and health risks of Chinese children, some errors will be made if inhalation exposure factors of the U.S. children are used directly. However, the existing data are insufficient to represent the characteristics of inhalation exposure and health risk assessment of Chinese children. Hence, it is urgent to launch a large-scale survey of children’s inhalation exposure factors nationwide. Through basic data accumulation and database establishment, children’s inhalation exposure factors fitting for Chinese children will be published. Meanwhile, much attention should be paid to differences of genders, activity levels, and urban and rural areas.

## 7. The Significance of Health Risk Assessment of Physical Activity in Polluted Environment

A wealth of data indicates that regular physical activity would provide benefits for physical and mental health of children and adolescents [61,62], improve life quality, reduce the risk of illness such as breast cancer and heart attack [63,64], and decrease the risk of premature morbidity and mortality. WHO advises that children and adolescents should at least participate in moderate-to-vigorous physical activity 60 min every day [65].

However, outdoor physical activity exposes children and adolescents to air pollutants (particulate matter, ozone, nitrogen oxides, etc.) which will negatively influence the physical activity behavior [66,67] and lead to adverse health problems such as cardiopulmonary, respiratory diseases [68,69,70] and other diseases such as lung cancer. Breathing polluted air during exercise can cause serious health problems [71]. That is to say, ambient air pollution in China poses a multifaceted health threat to outdoor physical activity participants [72]. We analyzed the characteristics of inhalation rate of different physical activities in different genders and ages, and get the result that we can adjust the intensity, exposure time and frequency of physical activity according to the different inhalation rates of different populations in physical activities, so as to minimize the negative influence on physical health of children and adolescents when they attend outdoor physical activity in the polluted environment.

Therefore, in the haze pollution, we can establish a relationship between activity patterns and environment variables which do not exceed the value of safe dose by changing physical activity variables (the most reasonable combination of the type of physical activity, intensity, time and frequency); children and adolescents can avoid hazards of pollution if they do physical activity at an appropriate level. That is to say, physical activity is not only an exposure factor, but also a critical inhalation exposure parameter for environmental assessment and Exercise Health Risk Assessment.

## 8. Conclusions

Physical activity is one of the most important and implementable methods of enhancing life quality and limiting premature mortality. The benefits of outdoor regular exercise are clear. Inhalation rate is the major inhalation exposure factor of children and adolescents’ Environmental Health Risk Assessment. Average daily breathing rates and time-activity patterns are of value in calculating inhalation intakes of contaminants whose concentration is determined daily. The average inhalation rates during active hours are higher than the rates during rest or sleep, and inhalation intakes are particularly sensitive to pollutant concentration in active or non-active periods, so physical activity is an important factor to influence the inhalation intake. In addition, there are also many factors which directly influence the magnitude and duration of inhalation intakes of particulate matter and other contaminants, such as intensity, time and time-activity patterns of physical activity. In the haze pollution, we can establish a relationship between activity patterns and environment variables by changing physical activity variables (the most reasonable combination of physical activity, intensity, time and frequency), which are below safe dose. Therefore, adolescents can avoid hazards of pollution and do physical activity at an appropriate level.

There is no children’s exposure factors handbook in China at present, and data of children inhalation rates and activity patterns are also limited, especially in exposure time. Existing data are insufficient to reflect exposure characteristics of residents in China. It does not improve the accuracy of exposure and health risk assessment results fundamentally and does not really meet the current and future environmental demand of health management. Thus, there is not enough theoretical support to provide emergency plan for youths in haze pollution.

Facing such a serious pollution in China, the government should pay special attention to the publication of children’s exposure factors handbooks. It is imperative to conduct nationwide researches and surveys on exposure factors of Chinese children and adolescents. The research should aim at promoting a new concept, which starts from understanding interactive relationship between the environment and human health, learning from the environment and health research techniques, and conducting the experiment of environmental health risk assessment related risk factors, e.g., controllable respiratory exposure parameters, so that children and adolescents can adapt to the malignant transformation of environment and actively participate in moderate physical activity in haze pollution, rather than stop sports activities passively and blindly. The new concept can provide theoretical guidance for making contingent plan of adolescents’ physical activity in haze pollution, and promote adolescents’ physical health effectively. The most important of all is to take physical activity as a critical composition of exposure factors to reduce the harm of environmental pollution. These factors could be considered for designing effective interventions of changing inactivity living habits and promoting physical health of adolescents.

## Figures and Tables

**Table 1:** Summaries of the surveys of physical activity pattern.

Name	Organizer	Date	Location	Sample	Age	Duration (Month)	Method
CAPS	CARB	1987–1988	California	1762	>12	12	24-h retrospective diaries
		1989–1990	California	1200	<12	12	24-h retrospective diaries
NHAPS	EPA	1992–1994	48 states of the U.S.	9386	The total population	24	24-h retrospective diaries
	EPA	2004–2008	48 states of the U.S.	23,028	The total population	24	GPS
	EPRI	1994–1995	The U.S.	1200	The total population	12	24-h retrospective diaries
CHAPS	EPA	1994–1995	Canada	2381	Thetotal population	9	24-h retrospective diaries

CARB: California Air Resources Board; EPA: United States Environmental Protection Agency; EPRI: Electric Power Research Institute (Duan et al., 2008) [36].

**Table 2:** Inhalation rate of children and adolescents in Shanghai in different physical activities.

Age (Year)	Gender	Rest (R) X ± S	Sedentary Behavior (SB) X ± S	Light Physical Activity (LPA) X ± S	Moderate Physical Activity (MPA) X ± S	Vigorous Physical Activity (VPA) X ± S
6	Male	0.22 ± 0.03	0.27 ± 0.01	0.33 ± 0.01	0.89 ± 0.05	1.33 ± 0.12
	Female	0.24 ± 0.03	0.29 ± 0.03	0.36 ± 0.01	0.95 ± 0.08	1.43 ± 0.15
7	Male	0.23 ± 0.04	0.28 ± 0.03	0.35 ± 0.05	0.94 ± 0.07	1.4 ± 0.13
	Female	0.25 ± 0.02	0.30 ± 0.01	0.37 ± 0.03	1.00 ± 0.08	1.49 ± 0.17
8	Male	0.25 ± 0.02	0.30 ± 0.03	0.38 ± 0.03	1.01 ± 0.05	1.51 ± 0.12
	Female	0.26 ± 0.01	0.310	0.39 ± 0.01	1.03 ± 0.11	1.55 ± 0.17
9	Male	0.267 ± 0.01	0.321	0.40 ± 0.04	1.07 ± 0.11	1.60 ± 0.12
	Female	0.270 ± 0.05	0.324	0.41 ± 0.02	1.08 ± 0.12	1.62 ± 0.21
10	Male	0.283 ± 0.03	0.340	0.42 ± 0.03	1.13 ± 0.07	1.70 ± 0.14
	Female	0.284 ± 0.04	0.341	0.42 ± 0.03	1.14 ± 0.19	1.71 ± 0.16
11	Male	0.31 ± 0.02	0.371	0.46 ± 0.04	1.24 ± 0.08	1.85 ± 0.13
	Female	0.30 ± 0.03	0.361	0.45 ± 0.02	1.21 ± 0.12	1.80 ± 0.17
12	Male	0.34 ± 0.05	0.404	0.51 ± 0.05	1.35 ± 0.15	2.02 ± 0.15
	Female	0.31 ± 0.11	0.374	0.47 ± 0.02	1.25 ± 0.13	1.87 ± 0.18
13	Male	0.36 ± 0.04	0.429	0.54 ± 0.03	1.43 ± 0.17	2.14 ± 0.20
	Female	0.32 ± 0.02	0.384	0.48 ± 0.04	1.28 ± 0.16	1.92 ± 0.15
14	Male	0.37 ± 0.05	0.445	0.57 ± 0.04	1.48 ± 0.17	2.22 ± 0.12
	Female	0.31 ± 0.04	0.390	0.49 ± 0.07	1.30 ± 0.15	1.95 ± 0.19
15	Male	0.38 ± 0.02	0.460	0.58 ± 0.05	1.54 ± 0.12	2.30 ± 0.16
	Female	0.33 ± 0.05	0.392	0.49 ± 0.04	1.31 ± 0.15	1.96 ± 0.18
16	Male	0.38 ± 0.07	0.461	0.58 ± 0.08	1.54 ± 0.19	2.31 ± 0.22
	Female	0.33 ± 0.01	0.392	0.49 ± 0.06	1.31 ± 0.12	1.96 ± 0.20
17	Male	0.39 ± 0.03	0.466	0.58 ± 0.05	1.55 ± 0.19	2.33 ± 0.18
	Female	0.33 ± 0.08	0.394	0.49 ± 0.08	1.31 ± 0.14	1.97 ± 0.16
18	Male	0.41 ± 0.02	0.486	0.61 ± 0.07	1.62 ± 0.18	2.43 ± 0.22
	Female	0.31 ± 0.01	0.368	0.46 ± 0.05	1.23 ± 0.13	1.84 ± 0.14
19	Male	0.40 ± 0.03	0.480	0.60 ± 0.07	1.60 ± 0.11	2.40 ± 0.25
	Female	0.30 ± 0.06	0.362	0.45 ± 0.06	1.21 ± 0.12	1.81 ± 0.23
20	Male	0.39 ± 0.05	0.467	0.58 ± 0.07	1.56 ± 0.17	2.34 ± 0.27
	Female	0.30 ± 0.01	0.363	0.45 ± 0.05	1.21 ± 0.12	1.81 ± 0.16

**Table 3:** Long-term inhalation rates of Chinese children and adolescents.

Age (Year)	Male		Female	
	Sample	Inhalation Rate (m^3^/d)	Sample	Inhalation Rate (m^3^/d)
1–2	35	4.7 ± 0.15	34	5.4 ± 0.16
3–5	168	5.9 ± 0.26	124	6.4 ± 0.42
6–8	161	9.1 ± 0.42	155	8.1 ± 0.67
9–11	193	10.6 ± 0.88	171	9.5 ± 0.91
12–14	206	12.2 ± 0.69	239	10.6 ± 1.02
15–18	239	13.5 ± 1.23	162	10.8 ± 0.88

**Table 4:** Short-term inhalation rates (m^3^/h) at different activity levels of Chinese children and adolescents.

Age (Year)	Gender	Rest	Sedentary	Light Intensity	Moderate Intensity	High Intensity	Extremely High Intensity
<6	Male	0.14 ± 0.02	0.17 ± 0.04	0.29 ± 0.04	0.57 ± 0.03	0.86 ± 0.07	1.43 ± 0.1
	Female	0.14 ± 0.05	0.17 ± 0.08	0.28 ± 0.05	0.56 ± 0.06	0.84 ± 0.05	1.40 ± 0.12
6–18	Male	0.29 ± 0.05	0.35 ± 0.29	0.59 ± 0.07	1.18 ± 0.1	1.77 ± 0.13	2.94 ± 0.23
	Female	0.28 ± 0.04	0.34 ± 0.04	0.57 ± 0.03	1.14 ± 0.09	1.70 ± 0.12	2.84 ± 0.36
